# A Novel Nanocomposite as an Efficient Adsorbent for the Rapid Adsorption of Ni(II) from Aqueous Solution

**DOI:** 10.3390/ma10101124

**Published:** 2017-09-22

**Authors:** Xiaotao Zhang, Ximing Wang, Zhangjing Chen

**Affiliations:** 1 College of Science, Inner Mongolia Agricultural University, Hohhot 010018, China; lianzixiaotao@163.com; 2 College of Material Science and Art Design, Inner Mongolia Agricultural University, Hohhot 010018, China; 3 Department of Sustainable Biomaterials, Virginia Tech University, Blacksburg, VA 24061, USA; chengo@vt.edu

**Keywords:** sulfhydryl-lignocellulose, montmorillonite, nanocomposite, Ni(II), adsorption

## Abstract

A sulfhydryl-lignocellulose/montmorillonite (SLT) nanocomposite was prepared using a chemical intercalation reaction. The SLT nanocomposite was characterized by Fourier Transform Infrared Spectroscopy (FTIR), X-Ray Diffraction (XRD), Scanning Electron Microscope (SEM), and Transmission Electron Microscopy (TEM), the results demonstrated that an intercalated-exfoliated nanostructure was formed in the SLT nanocomposite. Batch experiments were conducted to optimize parameters such as SLT nanocomposite dosage, the initial concentration of Ni(II), solution pH, temperature, and time. The results indicated that the attractive adsorption capacity reached 1134.08 mg/g with 0.05 g of SLT at an initial concentration of Ni(II) of 700 mg/L, solution pH of 5.5, adsorption temperature of 50 °C, and adsorption time of 40 min, meanwhile, the Ni(II) adsorption capacity significantly decreased with the increase in ionic strength. The pseudo-second order kinetic model could describe the whole adsorption process well, and the isotherm adsorption equilibrium conformed to the Freundlich model. The adsorption mechanism of SLT was also discussed by means of FTIR and Energy-Dispersive X-Ray (EDX). Dramatically, the introduction of sulfhydryl achieves the increased activated functional groups content of SLT nanocomposite, leading to remarkably higher adsorption amount on Ni(II). The desorption capacity of SLT was dependent on parameters such as HNO_3_ concentration, desorption temperature, and ultrasonic desorption time. The satisfactory desorption capacity and desorption efficiency of 458.21 mg/g and 40.40% were obtained at an HNO_3_ concentration, desorption temperature, and ultrasonic desorption time of 0.4 mol/L, 40 °C, and 30 min, respectively. The regeneration studies showed that the adsorption capacity of SLT was consistent for four cycles without any appreciable loss and confirmed that the SLT was reusable. Owing to such outstanding features, the novel SLT nanocomposite proved the great potential in adsorption for Ni(II) removal from aqueous solution, and exhibited an extremely significant amount of Ni(II), compared to pristine lignocellulose/montmorillonite and the conventional spent adsorbents.

## 1. Introduction

Heavy metal pollution arising from industrial and technological activities, i.e., metallurgy, printing, storage batteries, and rubber, plastic, aerospace, mineral, and pigment production is a global problem of worldwide concern [1,2]. Divalent nickel Ni(II), within permissible limits, is an essential heavy metal for living organisms. The tolerance limit in drinking water is 0.01 mg/L, and for industrial wastewater this value is 2.0 mg/L [3]. Though Ni(II) is an essential micronutrient for aquatic organisms and humans, exposure to high concentrations of Ni(II) may cause cancer of the lungs, nose, and bones, and may have mutagenic effects leading to critical health problems [4]. Hence, the removal of Ni(II) from effluents is environmentally important. 

To decrease high concentrations of Ni(II) to within permissible levels, various traditional methods such as ion exchange, chemical precipitation, evaporation, biological treatment, reverse osmosis, and electrochemical processes have been utilized for the reduction of Ni(II) ions from aqueous solutions [5,6,7]. However, the large capital requirements and need to dispose of toxic sludge are major shortcomings of these approaches. Adsorption is an effective process, the use of various spent adsorbents has many advantages including the highly efficient extraction of metals (even from dilute concentrations), minimization of secondary wastes, and cost-effectiveness. Until now, a number of non-conventional and low-cost adsorbents have been used for the removal of Ni(II) such as activated carbon, chelating resins, zeolite, chitosan, and montmorillonite [8,9,10]. However, as the adsorption capacities of the above adsorbents are limited, methods to improve the adsorption performance of new adsorbents are still under development.

Lignocellulose (LC), the most plentiful natural biopolymer, has been reported for its potential adsorption of heavy metals as its molecules contain a large number of active phenolic, hydroxyl, carboxyl, and other active groups that can be used in electrostatic interaction and in coordination sites for heavy metal ions [11]. However, its weak polydispersity properties, poor reactivity, and low specific gravity has made it difficult to apply extensively. Therefore, to overcome these issues and heighten the adsorption effect, specific attention has been given to derivatization by immobilizing sulfhydryl onto the LC. Generally, the sulfhydryl group site (SH-) exhibits high affinities for binding with heavy metals because of its coordination and chelation [12,13]. Moreover, a sulfhydryl-lignocellulose (SL) derivative enjoys many unique properties such as water solubility, controllable biodegradation, adhesive properties, and selectivity of heavy metals. The introduction of sulfur also contributes to greater stability in acidic solutions due to the formation of crosslinks [14,15]. In recent times, it has become particularly attractive with respect to adsorption applications. Currently, expandable layered silicates (e.g., montmorillonite (MT) clay) are receiving considerable recognition. However, due to low affinity, swelling, and dispersed suspension properties in water, MT adsorbs heavy metal ions only onto the external broken-bonds surface in very small amounts. Furthermore, the literature has shown that modified MT displays a higher adsorption capacity than the original clay [16]. 

Polymer/clay nanocomposites, as a very promising alternative for the expansion of industrial and economic activities and the satisfaction of increasingly stringent conditions, have attracted considerable attention from academic researchers [17,18]. To our knowledge, there have been no studies on the adsorption capacity of Ni(II) using a sulfhydryl-lignocellulose/montmorillonite (SLT) nanocomposite. Hence, a novel SLT nanocomposite was synthesized in this work. Furthermore, its adsorption and desorption capacities with respect to Ni(II) were observed in detail. Each of the factors influencing adsorption and desorption, including SLT nanocomposite dosage, initial concentration of Ni(II), pH values, adsorption temperature, adsorption time, concentration of HNO_3_, desorption temperature, and ultrasonic desorption time were investigated. In addition, the adsorption kinetics and isotherms with respect to SLT were studied, and the mechanisms of Ni(II) adsorption discussed. Finally, exploratory research results on the recycling application of the SLT nanocomposite have offered a reference for Ni(II) removal, and its regeneration ability was evaluated through four adsorption/desorption cycles, showing good recycling.

## 2. Experimental

### 2.1. Materials and Reagents

Lignocellulose was obtained from Beijing Huaduo Biotech Ltd., Beijing, China. The cationic exchange capacity (CEC) of montmorillonite was 100 mequiv/100 g was ground and sieved to a 200-mesh size (purchased from the Zhejiang Feng Hong Clay Chemical Co., Huzhou, China). Ni(NO_3_)_2_·6H_2_O was purchased from the Shanghai Jinshan Chemical Co., Shanghai, China. Sulfuric acid was the guaranteed reagent and was obtained from Beijing Huaduo Biotech Ltd., Beijing, China. Sodium acetate and glacial acetic acid were purchased from Hangzhou Chemical Reagent Co., Hangzhou, China. All other chemicals and reagents used in this study were purchased from Beijing Huaduo Biotech Ltd., Beijing, China, and were of analytical grade without further purification. All solutions were prepared using deionized water.

### 2.2. Preparation of Sulfhydryl-Lignocellulose (SL)

Dried lignocellulose powder (15 g) was mixed together with a mixture of 68 mL thioglycolic acid, 40 mL acetic anhydride, 30 mL acetic acid, and 2 mL sulfuric acid. The reaction mixture was stirred thoroughly and continuously at 40 °C for 45 h. Next, the suspension was washed several times with deionized water to provide a neutral pH and was then filtered. The treated SL was soaked in 2% NaOH for 10 h and the resultant solution was filtered and washed once. Then, the filter cake followed, being dissolved in 2% HCl for 2 h. The grey-green precipitate was separated by filtration, washed three times with deionized water again, and then dried under vacuum at 48 °C for 10 h to obtain the SL products. The obtained adsorbents were stored in dark.

### 2.3. Preparation of the Sulfhydryl-Lignocellulose/Montmorillonite (SLT) Nanocomposite

SL (4.0 g) was dissolved in 20% NaOH solution (weight of SL (g):volume of NaOH (mL) = 1:30) in batches, after being magnetically stirred, forming a uniform SL-NaOH suspension. Then, a suspension of MT (1.0 g suspended in 30 mL of deionized water) was stirred (500 rpm) for 0.5 h, followed by the addition of the SL-NaOH suspension. The temperature was then heated to 50 °C by stirring for 4.5 h. The reaction mixtures were centrifuged and washed with an acid solution until the pH of the supernatant reached 7. After that, the SLT was vacuum-dried at 105 °C (DZF-6210, Shanghai Yiheng Scientific Instrument Co., Ltd. Shanghai, China) for 10 min until the weight was stable. All samples were ground and sieved to a 200-mesh size. 

### 2.4. Standard Work Curve of Ni(II) Ions

Ni(II) standard solution was prepared by dissolving 0.4942 g Ni(NO_3_)_2_·6H_2_O in deionized water with addition of 2 mL concentrated sulfuric acid. 1 mL of prepared solution consists 0.10 mg Ni(II). The solution was stable for 10 days. Ni(II) working standard solution was prepared by dilution of 10 mL of basic standard solution in a volumetric flask (100 mL) and stirred. Dimethylglyoxime solution was done by dissolving dimethylglyoxime in ammonia after adding deionized water, and the solution was filtered. The solution had to be kept in dark bottle and it was stable for two weeks. 

In order to prepare calibration curve the following volumes of Ni(II) working standard solution 1.0, 2.0, 3.0, 4.0, 5.0, 6.0, 7.0, 8.0 and 10.0 mL were placed in nine clean volumetric flasks. Afterwards, 1 mL dimethylglyoxime solution, 2 mL saturated bromine water and 5 mL ammonia solution were added to each flask, respectively. And then add deionized water to the scale. The samples were mixed thoroughly. The prepared standards were ready to be used after 10 min but not later than after 30 min. Samples were transferred into the 1 cm of quartz color dish and scanned absorption in a double beam ultraviolet (UV)-visible spectrophotometer (TU-1901, Beijing Purkinje General Instrument Co., Ltd., Beijing, China) at the wavelength about 470 nm. The linear calibration curve was drawn as the function of absorption in different concentration of Ni(II) ions (mg/mL). The equation of the calibration curve was y = 0.00141x + 0.02618 and the coefficient correlation of the calibration curve was *R*^2^ = 0.9995. 

### 2.5. Adsorption Experiment

An amount of SLT (BS210S) was accurately weighted and added into a 50 mL Ni(II) solution with a measured concentration. The suspension was stirred at a uniform speed of 150 rpm in a thermostatic shaker (SHA-C) and pH was adjusted with a certain amount of sodium acetate/glacial acetic acid (NaOAc/HOAc) buffer solution using a pH meter (PB-10, Shanghai Youyi Instrument Co., Ltd., Shanghai, China). When the adsorption equilibrium was reached, the SLT nanocomposite was centrifuged at 5000 rpm for 5 min. The upper fluid was then taken to determine the residual concentration of Ni(II) by the dimethylglyoxime method. The absorbance of the wine-red-to-brown-colored Ni(II) complex using dimethylglyoxime was ready for use and scanned for absorption in the double beam ultraviolet (UV)-visible spectrophotometer at a wavelength corresponding to the maximum absorbance of about 470 nm. Spectroscopic grade standards were periodically checked during the experiment. Then, the concentrations of the samples were determined by using the linear regression equation (y = 0.00141x + 0.02618, *R*^2^ = 0.9995) for Ni(II) over a range of concentrations (100–1000 mg/L). NaNO_3_ and Mg(NO_3_)_2_ were used for ionic strength adjustment. The adsorption experiments were carried out using different SLT nanocomposite dosage, Ni(II) initial concentrations, pH, adsorption temperatures, and adsorption times. Taking into account the experimental errors and based on the average values, three independent replicates confirmed that the results of the Ni(II) removal experiments were reproducible in parallel under the same conditions and that the reproducibility of the results was within ±3%. The adsorption capacity of Ni(II) solution was measured from the following equation [19]:

(1) $$q_{t,1}=\frac{(C_{0}-C_{1})\times V_{1}}{m_{1}}$$

where *q_t_*_,1_ (mg/g) refers to the capacity of adsorption at time *t* (min). *C*_0_ and *C_t_*_,1_ (mg/L) refer to the Ni(II) initial concentration and final concentration at time *t* (min), respectively. *V*_1_ (mL) refers to the volume of Ni(II). *m*_1_ (g) is the mass of adsorbent. In calculating *q_t_*_,1_, no losses of Ni(II) ions to any other mechanism (volatilization, sorption to the glassware, degradation, etc.) were assumed.

### 2.6. Desorption and Regeneration Experiments

The Ni(II)-loaded SLT nanocomposite (0.05 g) was accurately weighted, transferred into various 50 mL desorption reagents, and put into an ultrasonic cleaning machine (KS-300EI, Qingdao Shengzhong Instrument Co., Ltd., Qingdao, China). When the desorption equilibrium was reached at a certain temperature, the suspension was centrifuged, thus determining the concentrations of the desorbed Ni(II) solution. The effects of different desorption agents and concentration, desorption temperatures, and ultrasonic desorption times were studied. The final Ni(II) concentrations in solution were analyzed. Taking into account the experimental errors and based on the average values, three independent replicates confirmed that the results of desorption experiments were reproducible in parallel under the same conditions and the reproducibility of the results was within ±3%. The desorption capacity of the Ni(II)-loaded SLT and the desorption efficiency were calculated according to the following Equations (2) and (3) [20].

(2) $$q_{t,2}=\frac{C_{t,2}\times V_{2}}{m_{2}}$$

(3) $$\mathrm{Desorption}(\%)=\frac{q_{t,2}}{q_{\max}}\times 100\%$$

where *q_t_*_,2_ (mg/g) refers to the desorption amount at time *t* (min). *C_t_*_,2_ (mg/L) refers to the concentration of Ni(II) in the desorbed solution at time *t* (min). *V*_2_ (mL) refers to the total volume of solution in desorption. *m*_2_ (g) refers to the mass of the adsorbent after adsorption of Ni(II). *q*_max_ (mg/g) refers to the maximum adsorption.

Repeated batch experiments were performed to examine the reusability of SLT for Ni(II). After the desorption equilibrium was completed, the suspension was separated from the adsorbent by centrifugation at 6000 rpm for 10 min, washed with deionized water to remove the remaining acid, and vacuum-dried in an oven (DZF-6210) at 50 °C for the next adsorption of Ni(II). The adsorption and desorption capacities of Ni(II) were determined and analyzed. The consecutive adsorption/desorption processes were performed five times.

### 2.7. Characterization

FTIR spectra were recorded in KBr pellets using a Thermo Nicolet Nexus™, spectrometer. XRD analyses of the powdered samples were performed using an X-ray power diffractometer with Cu anode (PAN Analytical Co., X’pert PRO, Almelo, The Netherlands), running at 40 kV and 30 mA, scanning from 4 to 18° at 3/min. SEM of the samples was performed using a HITACHI S-4800 microscope (Tokyo, Japan). EDX analysis was performed using energy dispersive X-ray spectroscopy (HITACHI S-4800, Tokyo, Japan). Before SEM observation, all samples were fixed on aluminum stubs and coated with gold. TEM image analysis of the samples was performed using a TEM (JEM-2010, Tokyo, Japan) at 200 kV.

## 3. Results and Discussion

### 3.1. FTIR Analysis of SLT

**Figure 1:**
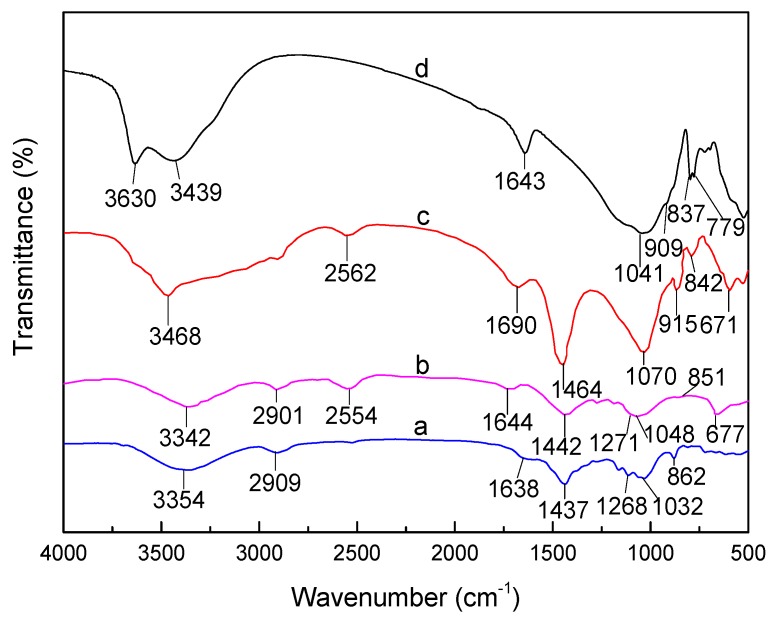
Fourier Transform Infrared Spectroscopy (FTIR) spectra of (a) purified montmorillonite (MT); (b) the sulfhydryl-lignocellulose/montmorillonite (SLT) nanocomposite; (c) sulfhydryl-lignocellulose (SL); and (d) purified lignocellulose (LC).

FTIR spectra of (a) purified SL, (b) LC, (c) SLT, and (d) MT are shown in Figure 1. Compared with the FTIR of LC, the adsorption bands were at 3354 cm^−1^ (Figure 1a) for the –OH bending vibration of H_2_O, at 2909 cm^−1^ for the C–H stretching on methyl and methylene, at 1638 cm^−1^ for the –OH bending vibration of H_2_O of LC, at 1437 cm^−1^ for the –C–O–C– symmetrical telescopic vibration, at 1268 cm^−1^ for the C–O symmetrical telescopic vibration, at 1032 cm^−1^ for the C–O–C and C–O stretching vibrations, and at 862 cm^−1^ for the stretching vibration of the aromatic and phenol C–H stretching. They all strengthened and shifted to wave numbers 3348 cm^−1^, 2914 cm^−1^, 1644 cm^−1^, 1442 cm^−1^, 1271 cm^−1^, 1048 cm^−1^, 851 cm^−1^, respectively (Figure 1b). In addition, strong characteristic absorption bands around 2554 cm^−1^ and 667 cm^−1^ were due to the sulfhydryl group (–SH) stretching vibration and C–S bending vibration appeared in SL (Figure 1b) [21,22]. This information from the FTIR spectra (Figure 1a,b) indicated that the surface groups of LC had been derived and new sulfhydryl groups had been formed. 

Compared with the FTIR spectra of (b) SL, and (d) MT, the adsorption bands at 3630 cm^−1^ and 3439 cm^−1^ assigned to the –OH stretching vibration of H_2_O of MT (Figure 1d) shifted to wave number 3468 cm^−1^ (Figure 1c). The characteristic adsorption band at 1643 cm^−1^ of MT (Figure 1d) and 1644 cm^−1^ of SL (Figure 1b) moved to 1690 cm^−1^ (Figure 1c). Simultaneously, the intensity of this adsorption band increased, which indicated that the –CO group stretching vibration of LC overlapped with the –OH bending vibration of H_2_O in MT. At the same time, the –COO^−^ group adsorption at 1442 cm^−1^ and a second –OH group adsorption at 1048 cm^−1^ of SL (Figure 1b) was observed on the FTIR spectra of SLT (Figure 1c) at 1464 cm^−1^ and 1070 cm^−1^, respectively. The adsorption bands of MT were due to the telescopic vibration of Al–O–H, Si–O and Mg–O–H at 909 cm^−1^, 837 cm^−1^ and 779 cm^−1^, respectively, which were obviously weakened in the spectra of the SLT nanocomposite (Figure 1c). Especially important were the characteristic adsorption bands around 2554 cm^−1^ and 667 cm^−1^, which corresponded to S–H; and the C–S stretching vibration strengthened and shifted to wave numbers 2562 cm^−1^ and 671 cm^−1^ in the SLT nanocomposite (Figure 1c). It was concluded from the above discussion that the SL was introduced into the interlayer space of MT, and the activated adsorption sites of SLT were not only on –COO^−^, but also on –OH, –SH, C=O, C–O–C, Si–O, Al–O and so on, which may have had a tremendous influence on the adsorption properties of the nanocomposite [23].

### 3.2. XRD Analysis of SLT

**Figure 2:**
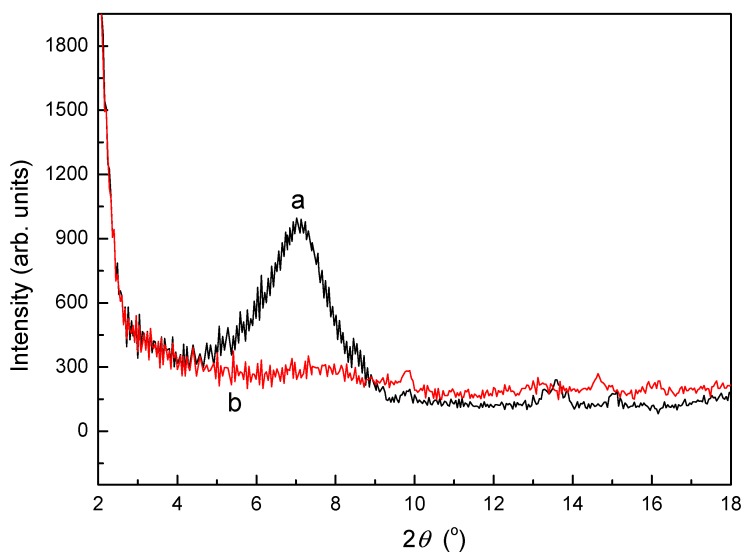
X-ray Diffraction (XRD) powder patterns of (a) MT; and (b) the SLT nanocomposite.

XRD is an effective method for determining the morphological features of adsorbents. Figure 2 shows the XRD images of (a) MT, and (b) the SLT nanocomposite. The XRD pattern of MT (Figure 2a) displayed a typical diffraction peak at 6.94°, responding to a basal spacing of 1.27 nm, which showed typical nanostructure features. However, after intercalation with SL, this peak shifted to a lower angle and even disappeared (Figure 2b). The XRD patterns indicated that SL had been intercalated into an MT interplayer. According to the results of the FTIR and XRD analysis, it can be conformed that a disordered intercalated–exfoliated structure may have formed in the SLT nanocomposite. 

### 3.3. SEM Analysis of SLT

**Figure 3:**
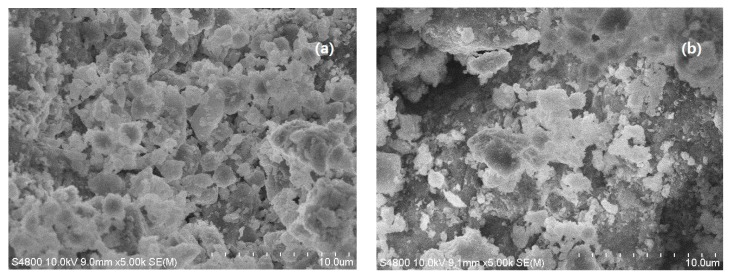
SEM images of (**a**) MT; and (**b**) the SLT nanocomposite.

The morphologies of (a) purified MT, and (b) SLT are shown in Figure 3 where it can be seen that MT showed small particles and a nonporous surface (Figure 3a); however, the intercalation of SL into MT resulted in large particles with a coarse porous surface (Figure 3b). The incorporation of SL produces numerous cavities and a relatively loose structure, which eventually leads to an increase in the contact areas and activated sites for the adsorption of Ni(II) ions. Therefore, these phenomena indicated that almost all of the SL was intercalated into the MT interlayer by destroying its crystalline structure, forming the intercalated and exfoliated SLT nanocomposite. This was in accordance with the results of the XRD patterns.

### 3.4. TEM Analysis of SLT

**Figure 4:**
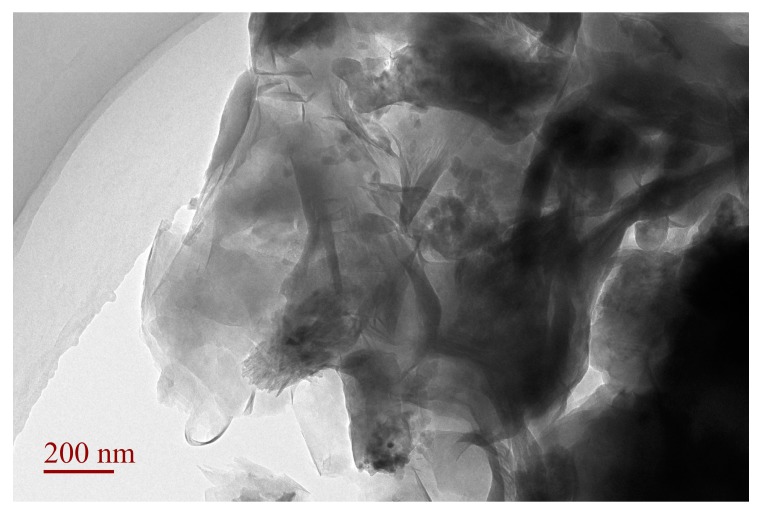
Transmission Electron Microscopy (TEM) images of the SLT nanocomposite.

More direct evidence of the morphology of the true nanocomposite was provided by TEM imaging. The combination of XRD patterns and TEM analysis is a powerful method when characterizing the microstructure of polymer/clay nanocomposites. TEM images of the SLT nanocomposite are shown in Figure 4. It was found that the stacks of multilayers of MT became thin and dispersive, which indicated that the dispersion of MT nano-platelets was achieved, and almost all SL materials were embedded with the destruction of the crystalline structure into MT interlayers. The polymer matrix was well dispersed in the nanolayers. This confirmed that the intercalated–exfoliated structure still existed in the SLT nanocomposite, as proven by the XRD analysis as described above.

### 3.5. Influencing Factors on Ni(II) Adsorption

#### 3.5.1. Effect of Adsorbent Dosage

**Figure 5:**
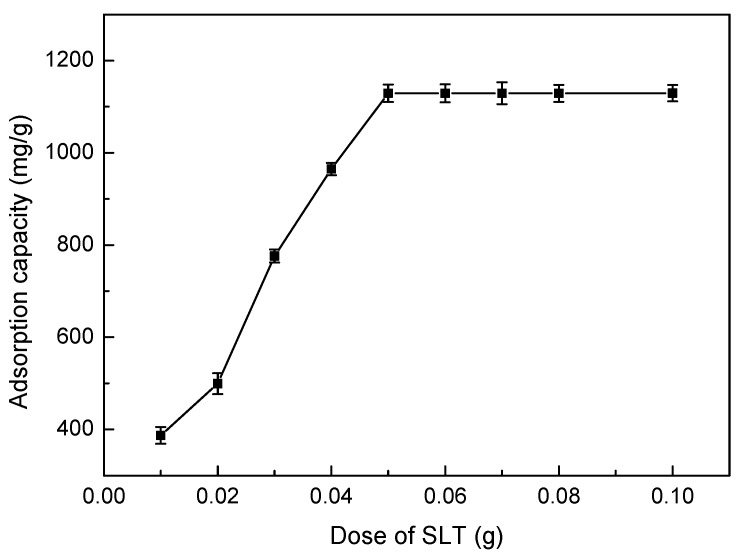
Effect of SLT dose on adsorption capacity of the SLT nanocomposite. (Adsorption experiments—sample dosage: 0.01–0.10 g; initial Ni(II) concentration: 700 mg/L; pH value: 5.5; temperature: 50 °C; adsorption time: 40 min).

The amount of adsorbent is an important parameter because it determines the adsorption capacity of an adsorbent for a given initial concentration of the adsorbate. The effects of different SLT dosage on removal of Ni(II) was carried out, and the results have been shown in Figure 5. The amount of SLT was varied from 0.01 g to 0.10 g while all the variables such as initial concentration of Ni(II), pH values, adsorption temperature, adsorption time were kept constant. It is clear from Figure 5 that the adsorption capacity of Ni(II) increased rapidly over range of 0.01–0.05 g of SLT nanocomposite dissolved in Ni(II) aqueous solution. Beyond 0.05 g of SLT, the adsorption capacity of Ni(II) ions remains unchanged. This is due to greater availability of active sites. Thus, the dose of SLT nanocomposite was fixed to 0.05 g for the subsequent adsorption experiments. 

#### 3.5.2. Effect of Ni(II) Initial Concentration

**Figure 6:**
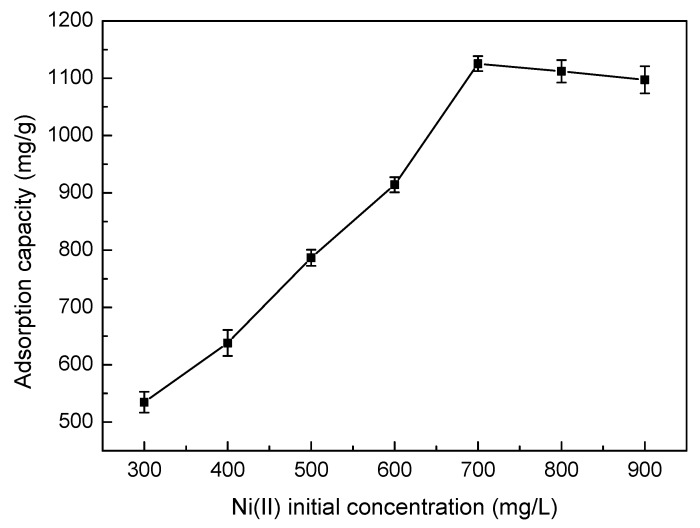
Effect of Ni(II) concentration on adsorption capacity of the SLT nanocomposite. (Adsorption experiments—sample dosage: 0.05 g; initial Ni(II) concentration: 300–900 mg/L; pH value: 5.5; temperature: 50 °C; adsorption time: 40 min).

The effects of different Ni(II) initial concentrations on SLT adsorption capacity are shown in Figure 6. It can be seen that the adsorption capacity trend of the SLT nanocomposite towards Ni(II) first increased rapidly, then remained nearly stable when the Ni(II) initial concentration was increased. This was likely due to an increase in the amount of Ni(II) ions, which led to the increase of collision times between the Ni(II) ions and the active adsorption sites on the SLT, hence increasing adsorption capacity. When Ni(II) concentration was further increased, the adsorption capacity remained stable due to the saturation of active adsorption sites. Therefore, Ni(II) with an initial concentration of 700 mg/L was chosen as the ideal initial concentration condition.

#### 3.5.3. Effect of pH

**Figure 7:**
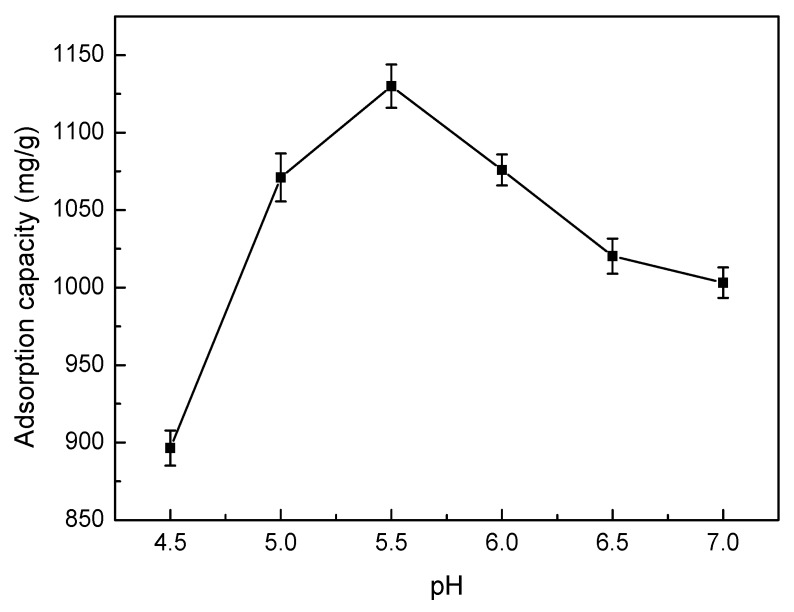
Effect of pH on the adsorption capacity of the SLT nanocomposite. (Adsorption experiments—sample dosage: 0.05 g; initial Ni(II) concentration: 700 mg/L; pH value range: 4.5–7.0; temperature: 50 °C; adsorption time: 40 min).

The pH value of the Ni(II) solution is an important factor for determining the adsorption of solutes. The influence of pH values on the SLT adsorption capacity is shown in Figure 7. This result indicated that the trend on adsorption capacity of SLT of Ni(II) exhibited an increase at first, followed by a decrease with increasing pH. When the pH was 5.5, the adsorption capacity reached the maximum amount of 1129.88 mg/g. This result can be accounted for as follows: when the pH was less than 5.5, the main reactive functional groups in SLT were –COOH, –OH and –SH; Ni(II) sorption through the exchange of ions was favored at low pH values, especially when the sorption rate was largely controlled by ion exchange rather than by complexation. As the pH increased, the anion group concentration (–COO^−^) increased, and the coordination and chelation ability of Ni(II) with SLT gradually increased. However, when the pH was higher than 5.5, Ni(II) could react with a basic pH regulator, which resulted in facile complexation or precipitation and therefore a reduction in adsorption capacity [24]. It was determined that the optimum pH for adsorption was 5.5. 

#### 3.5.4. Effect of Adsorption Temperature

**Figure 8:**
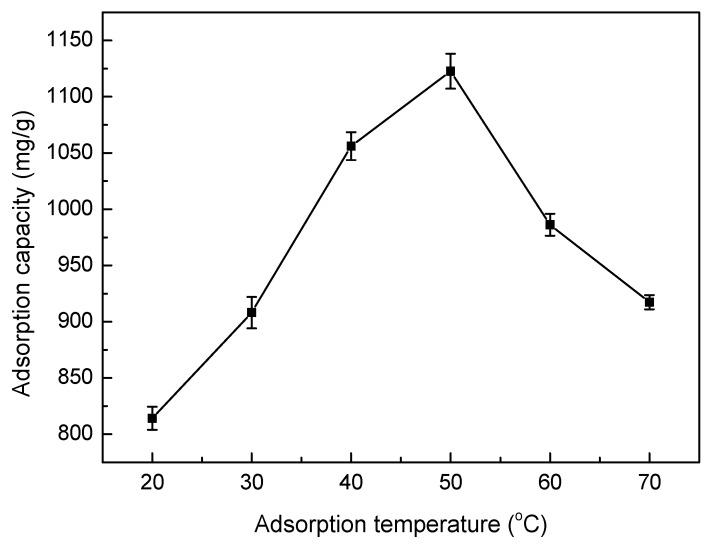
Effect of adsorption temperature on the adsorption capacity of the SLT nanocomposite. (Adsorption experiments—sample dosage: 0.05 g; initial Ni(II) concentration: 700 mg/L; pH value: 5.5; temperature range: 20–70 °C; adsorption time: 40 min).

Figure 8 shows the relationship between the different temperatures and the adsorption capacity of Ni(II) using the SLT nanocomposite. It can be seen that the adsorption capacity towards Ni(II) first increased, then dropped with a rise in temperature. This result can most likely be attributed to the enhanced activity of SLT molecules with an increase in adsorption temperature, which was caused by the disruption of intermolecular hydrogen bonding interactions between the molecular chains with the acceleration of molecular thermal motion. With an increased number of activated molecules, the interaction between Ni(II) and SLT was also enhanced, which was conducive to increasing the absorption capacity. However, continued heating was shown to lead to the decomposition of SLT with damage to the three-dimensional structures. It was found that the higher temperature was to the advantage of adsorption and that adsorption was an endothermic reaction. Therefore, an adsorption temperature of 50 °C was chosen as the ideal condition.

#### 3.5.5. Effect of Adsorption Time

**Figure 9:**
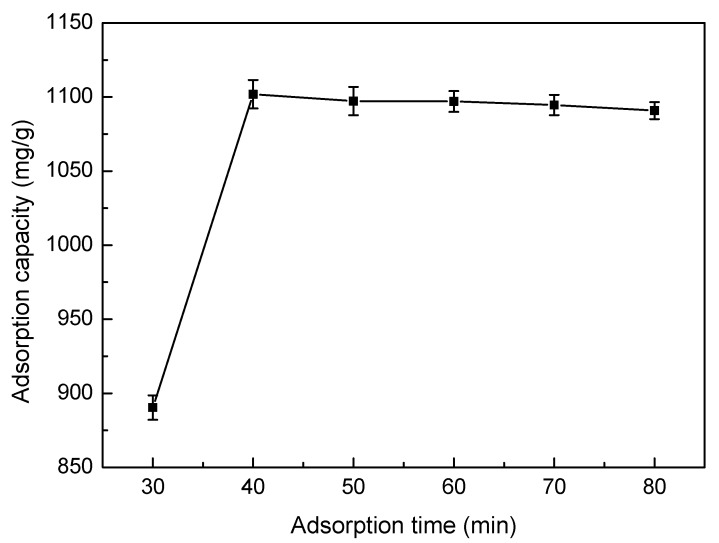
Effect of adsorption time on adsorption capacity of the SLT nanocomposite. (Adsorption experiments—sample dosage: 0.05 g; initial Ni(II) concentration: 700 mg/L; pH value: 5.5; temperature: 50 °C; adsorption time range: 30–80 min).

The effects of different adsorption times on SLT adsorption capacity are shown in Figure 9. As indicated in Figure 9, when contact time was prolonged, the trend of adsorption capacity of the SLT towards Ni(II) increased rapidly at first, then remained stable. This may be considered as a result of Ni(II) being introduced to the adsorbent surface for a short contact time, which was then followed by a spread into the adsorbent micropores, and finally the formation of a complex with the active sites of the adsorbent, thus resulting in a sharp adsorption equilibrium. Therefore, in this study, the optimum adsorption time was selected as 40 min.

#### 3.5.6. Effect of Ionic Strength

**Figure 10:**
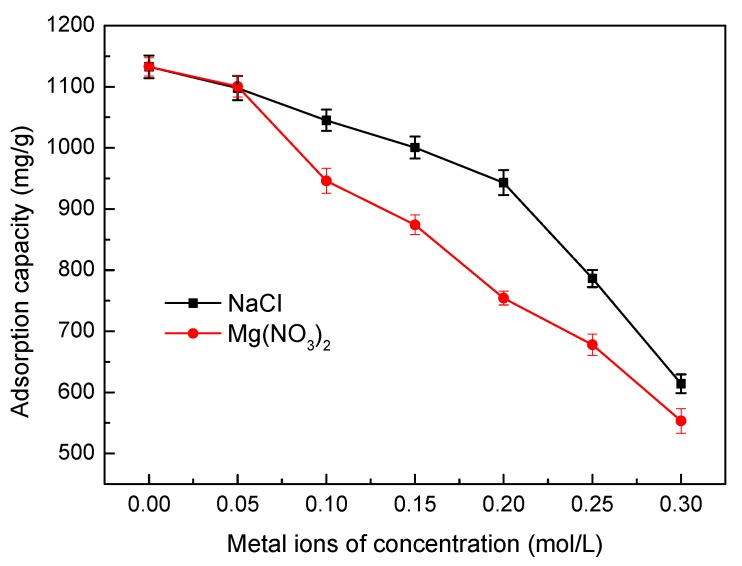
Effect of ionic strength on adsorption capacity of the SLT nanocomposite. (Adsorption experiments—sample dosage: 0.05 g; initial Ni(II) concentration: 700 mg/L; pH: 5.5; temperature: 50 °C; adsorption time: 40 min).

To evalutate the effect of ionic strength on the adsorption of Ni(II), adsorption experiments were performed by adding NaCl and Mg(NO_3_)_2_ at different concentrations. The results are presented in Figure 10. It can be seen from Figure 10 that increasing the ionic strength from 0 mol/L to 0.30 mol/L leads to a significant decrease in Ni(II) adsorption. The results may be arrtibuted to the following factors. In the course of adsorption process, two different surface complexes can be formed, inner sphere and out sphere [25]. In the inner sphere surface complexes, the adsorbed molecules or ions and the surface activated functional groups formed covalent bonds, however in the outer sphere surface complexes, no covalent bonds formed. Therefore, other interactions concluding electrostatic attraction, hydrophobic attraction and hydrogen bonding are responsible for the sorption. An inner sphere surface complex is insensitivity to the ionic strength. The decrease in adsorption with the increase in ionic strength has been explained an outer sphere surface complex. The ionic-strength-dependent adsorption may be owing to the formation of outer sphere complexes through cation exchange at activated sites resulting from the interactions between metal ions and a negative surface charge. In summary, increasing the Na^+^ and Mg^2+^ concentration can increase the competition with Ni(II) for the functional sites on SLT nanocomposite, reducing the adsorption capacity. Moreover, the effect of Mg^2+^ is more apparent than that of Na^+^ because Mg^2+^ can attract more negative charges on SLT, displaying a higher inhibition of Ni(II) adsorption.

### 3.6. Adsorption Kinetics

To study the potential rate-controlling steps of Ni(II) adsorption, four different kinetic models were used to fit the experimental data: the pseudo-first-order model; the pseudo-second-order model; intraparticle diffusion; and the Elovich kinetic model, which are expressed as Equations (4)–(7) [26,27]:

(4) $$\log(q_{e}-q_{t})=\log q_{e}-\frac{k_{1}t}{2.303}$$

(5) $$\frac{t}{q_{t}}=\frac{1}{k2q_{e}{}^{2}}+\frac{t}{q_{e}}$$

(6) $$q_{t}=k_{i}t^{0.5}$$

(7) $$q_{t}=\frac{1}{\beta }\ln(\alpha \beta )+\frac{1}{\beta }\ln t$$

where *q_e_* and *q_t_* are the amounts of Ni(II) adsorbed (mg/g) at equilibrium and at time *t* (min), respectively; *k*_1_ (min^−1^) is the pseudo-first-order rate constant; *k*_2_ [g·(mg/min)^−1^] is the rate constant of the pseudo-second-order adsorption kinetic equation; *k_i_* (mg·(g·min^0.5^)^−1^) is an intraparticle diffusion rate constant; *α* [mg·(g·min)^−1^] is the initial adsorption rate; and *β* (g/mg) is related to the surface coverage and activation energy for chemisorption. It is well-known that the pseudo-first-order model assumes that the rate of adsorption is directly proportional to the difference between the adsorption capacity with time and the saturation capacity at equilibrium. In addition, the pseudo-second-order model assumes that the rate of adsorption depends on the square of the difference between the amount of metals adsorbed at the interface with time *t* and the adsorption capacity at equilibrium. The intraparticle diffusion model of Weber and Morris assumes that intraparticle diffusion is a rate-determining step of the adsorption process, and the Elovich model assumes that the adsorption sites are heterogeneous and display a variety of activation energies during the adsorption process. 

**Figure 11:**
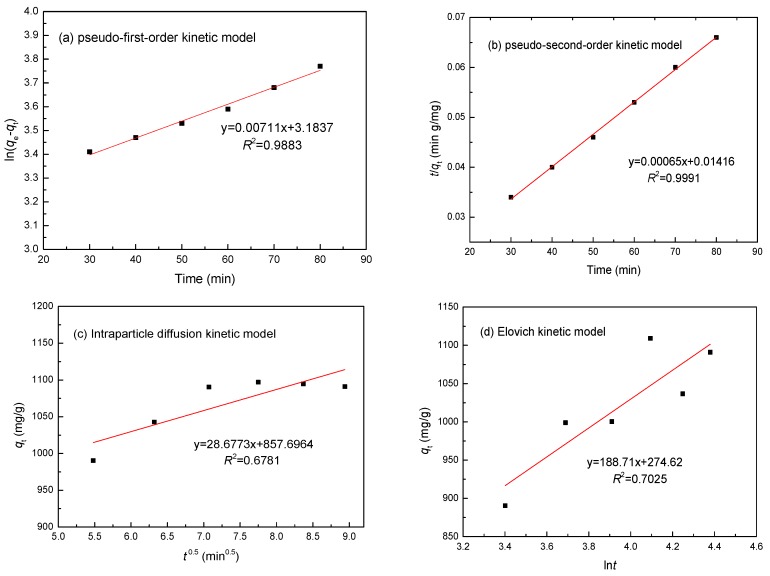
(**a**) Pseudo-first-order; (**b**) pseudo-second-order; (**c**) Intraparticle diffusion model; and (**d**) and Elovich kinetic models for the adsorption of Ni(II) ions by SLT.

The fits of these four models were checked by each linear plot of ln(*q_e_* − *q_t_*) versus *t* (Figure 11a), (*t*/*q_t_*) versus *t* (Figure 11b), *q_t_* versus *t*^0.5^ (Figure 11c), and *q_t_* versus ln*t* (Figure 11d), respectively. The *R*^2^ and constant values for the four adsorption kinetic models were calculated and are given in Table 1. According to Figure 11, the calculated kinetic model parameters in Table 1, and a comparison of the experimental equilibrium adsorption capacity for the adsorption of Ni(II) onto the SLT nanocomposite, the results suggested an ideal fit to the pseudo-second-order model with an extremely high *R*^2^ (0.9991). A good agreement could further be supported by the similar values of the calculated and experimental values of *q_e_*. Therefore, it was obvious that chemical adsorption should be the rate-limiting step of the adsorption of Ni(II) onto the SLT nanocomposite.

### 3.7. Adsorption Isotherm

Adsorption isotherm models are commonly used to describe adsorption and investigate its mechanisms. Hence, four Langmuir, Freundlich, Temkin and Dubinin–Radushkevich isotherm models were applied to analyze the adsorption equilibrium experimental data obtained for the adsorption of Ni(II) on the SLT nanocomposite. Equations (8)–(12) are given as follows [28,29]:

(8) $$\frac{C_{e}}{q_{e}}=\frac{1}{K_{L}q_{\max}^{}}+\frac{C_{e}}{q_{\max}}$$

(9) $$\ln q_{e}=\ln K_{f}+\frac{1}{n}\ln C_{e}$$

(10) $$q_{e}=\frac{RT}{b_{t}}\ln\alpha_{t}+\frac{RT}{b_{t}}\ln C_{e}$$

(11) $$\ln q_{e}=\ln q_{\max}-B\varepsilon^{2}$$

(12) $$\varepsilon =RT\ln(1+\frac{1}{C_{e}})$$

where *K_L_* (L/mg) is the Langmuir constant related to the adsorption capacity; *q*_max_ (mg/g) is the monolayer saturation adsorption capacity; 1/*n* is the value used to indicate the heterogeneity of the interface; *K_f_* is the Freundlich constant; *C_e_* (mg/L) is the concentration of metal ions at equilibrium; *q_e_* (mg/g) is the adsorption capacity at equilibrium; *R* is the ideal gas constant (8.314 J mol^−1^ K^−1^); *T* (K) is the absolute temperature of the adsorption process; *α_t_* (L/g) and *b_t_* (J/mol) are Temkin isotherm constants; *B* is a constant; and *ε* is the Polanyi potential, which can be calculated using Equation (11). Theoretically, the Langmuir isotherm assumes that the surface of the adsorbent contains homogeneous binding sites with identical adsorption energies and has no interactions with the adsorbed molecules. The Freundlich isotherm assumes adsorption on a heterogeneous surface in nature with a non-uniform distribution of adsorption heat on the surface. The Temkin isotherm assumes that the heat of adsorption decreases linearly as adsorption increases and predicts a uniform distribution of binding energies over the population of the surface binding adsorption sites. The Dubinin–Radushkevich model is used to determine the characteristic porosity and the apparent free energy of adsorption. 

The essential characteristics of the Langmuir isotherm can be represented according to a dimensionless equilibrium parameter (*R_L_*) based on the following equation [30]:

(13) $$R_{L}=\frac{1}{1+K_{L}C_{0}}$$

where *K_L_* (L/mg) is the Langmuir adsorption constant; and *C*_0_ is the optimal concentration of Ni(II) ions. The value of *R_L_* indicates the nature of the isotherm as unfavorable (*R_L_* > 1), linear (*R_L_* = 1), favorable (0 < *R_L_* < 1), or irreversible (*R_L_* = 0).

**Figure 12:**
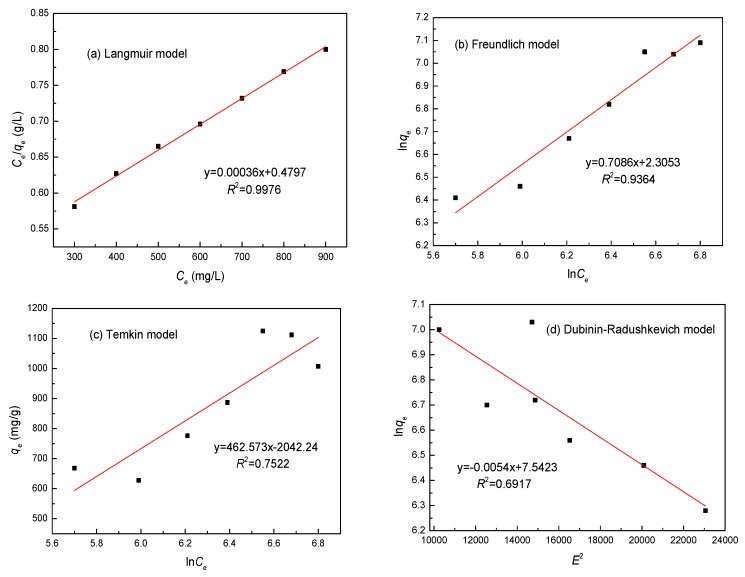
(**a**) The Langmuir; (**b**) Freundlich; (**c**) Temkin; and (**d**) Dubinin-Radushkevich isotherm models for the adsorption of Ni(II) ions by SLT.

Comparisons of these isotherm models for the adsorption of Ni(II) onto SLT were performed by comparing each linear plot of *C_e_*/*q_e_* versus *C_e_* (Figure 12a), ln*q_e_* versus ln*C_e_* (Figure 12b), *q_e_* versus ln*C_e_* (Figure 12c), and ln*q_e_* versus *ε*^2^ (Figure 12d). The calculated constants are listed in Table 2. From Figure 12 and Table 2, it is clear that the coefficients *R*^2^ of the linear form of the Langmuir model (0.9976) were closer to 1 than that of the other models. In addition, the maximum monolayer adsorption capacity (*q*_max_) value calculated from the Langmuir model was 1120.33 mg/g, which was almost the same as in the experimental data (1134.08 mg/g). This result may be due to the homogeneous distribution of the activated sites on the surface of the SLT. Furthermore, the value of *R_L_* for the Langmuir isotherm was between 0 and 1, and the Freundlich constant 1/*n* was smaller than 1, indicating a favorable process. Obviously, the Langmuir model was much better for describing the adsorption of Ni(II) onto the SLT nanocomposite. It is known that the Langmuir model corresponds to a dominant electrostatic attraction, ion exchange, coordination, and chelation mechanism. This meant that the adsorption process involved physical adsorption and the monolayer coverage chemical complexation at the interface and the outer heterogeneous surface to SLT. The *q*_max_ values of Ni(II) on the SLT nanocomposite were compared with those of other adsorbents and are listed in Table 3. It can be concluded that the *q*_max_ values of other materials were much lower than those of the SLT nanocomposite. Consequently, the high adsorption capacity in this paper revealed that the SLT nanocomposite can be employed as an excellent novel adsorbent to remove Ni(II) from aqueous solutions.

### 3.8. Desorption and Regeneration

To obtain a better understanding of the adsorption mechanism for Ni(II) onto SLT, and considering that reusing the adsorbent keeps the processing costs down and allows the possibility for recovering heavy metals extracted from wastewater, sequential desorption was carried out after the adsorption experiments in this test. Then, the effects of HNO_3_ concentration, desorption temperature, and ultrasonic desorption time on desorption efficiency of SLT were discussed in detail.

**Figure 13:**
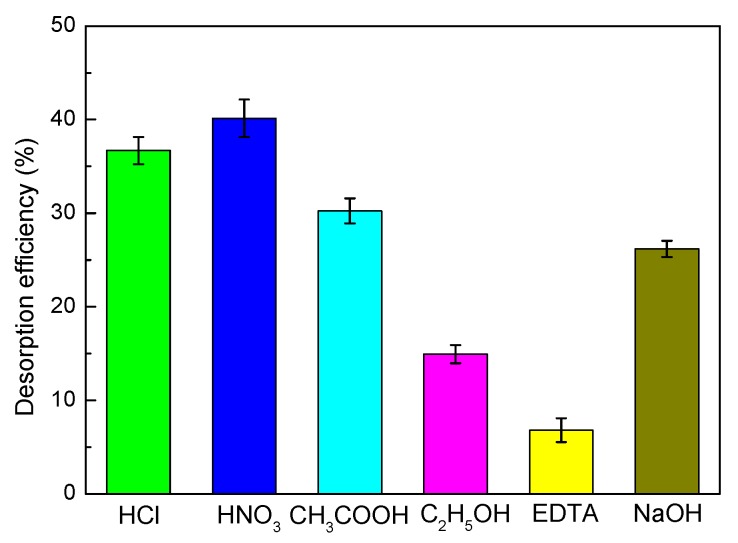
Effect of different desorption agents on desorption efficiency of the SLT nanocomposite. (Desorption experiments—sample dosage: 0.05 g; desorption agent concentration: 0.4 mol/L; desorption temperature: 40 °C; desorption time: 30 min).

First, a series of desorption and regeneration experiments were conducted using HCl, HNO_3_, CH_3_COOH, C_2_H_5_OH, ethylene diamine tetraacetic acid (EDTA), and NaOH as the desorbing eluents to investigate their effects on desorption (Figure 13). From Figure 13, it can be clearly seen that EDTA (6.8%) was almost useless for desorbing the bonded Ni(II) ions from the SLT. The desorption efficiency of C_2_H_5_OH was slightly higher than NaOH, but still displayed lower desorption efficiency when compared with the acid solutions. Among the three acidic desorption eluents, HCl, HNO_3_, and CH_3_COOH, HNO_3_ was found to be a good regenerating solution for Ni(II)-loaded SLT. This result revealed that the surface of SLT was protonated by H^+^ ions under acidic conditions, and that electrostatic interactions occurred between H^+^ and the activated sites, thereby leading to the desorption of positively charged Ni(II). Furthermore, ion-exchange, electrostatic attraction, coordination and chelation mechanisms were determined, and HNO_3_ (40.13%) could be an effective desorption eluent for the regeneration of Ni(II)-loaded SLT in this work.

**Figure 14:**
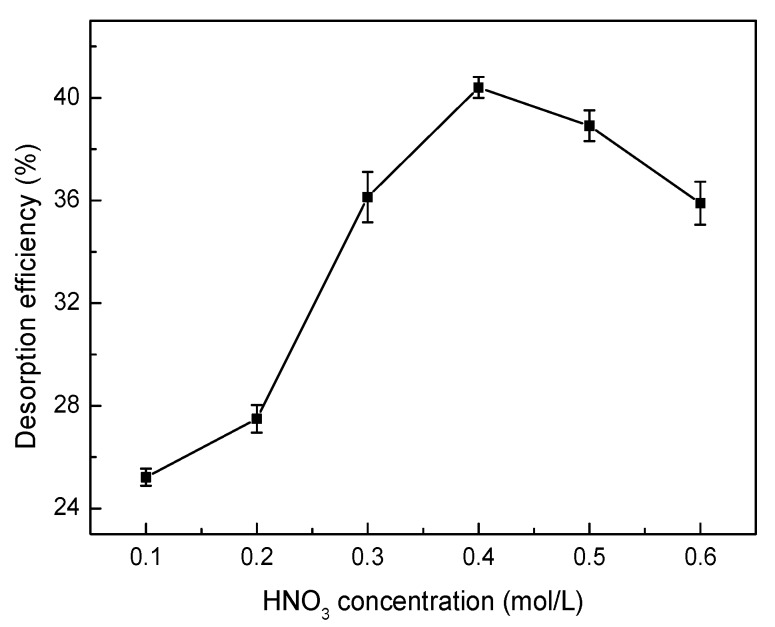
Effect of HNO_3_ concentration on desorption efficiency. (Desorption experiments—sample dosage: 0.05 g; desorption agent concentration: 0.1–0.6 mol/L; desorption temperature: 40 °C; desorption time: 30 min).

Second, Figure 14 presents the effects of different HNO_3_ concentrations on the desorption efficiency of SLT. As shown, the desorption efficiency initially increased, then decreased with increasing HNO_3_ concentration, possibly because the accumulated H^+^ concentration increased the concentration gradients of Ni(II) and H^+^, which further facilitated the desorption of loaded-Ni(II). The relatively high desorption efficiency (40.4%) of HNO_3_ at a concentration of 0.4 mol/L suggested that the adsorption of Ni(II) onto SLT was carried out partially via electrostatic attraction and ion exchange, which substantiated the results on pH values with respect to adsorption.

**Figure 15:**
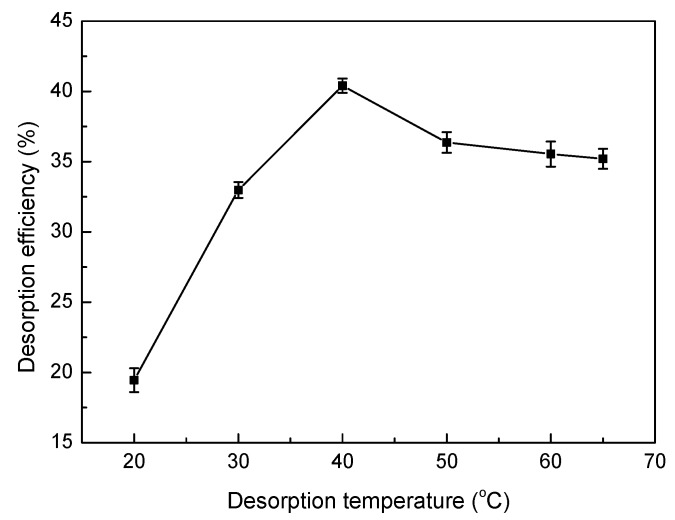
Effect of desorption temperature on desorption efficiency. (Desorption experiments—sample dosage: 0.05 g; desorption agent concentration: 0.4 mol/L; desorption temperature: 20–65 °C; desorption time: 30 min).

Third, the effects of different desorption temperatures on the desorption efficiency are shown in Figure 15. The desorption efficiency first increased, then slightly decreased with increasing temperature. This fluctuation could be attributed to the fact that increasing temperature may enhance the adsorption activity and efficiency of the reaction sites on the surface of the SLT nanocomposite. H^+^ and Ni(II) may compete with each other for the activated sites, leading to an increase in desorption efficiency. In addition, a higher desorption temperature may impair the adsorption efficiency of the active sites, thus producing a detrimental effect on the desorption process, which further supports the results of adsorption temperature. 

**Figure 16:**
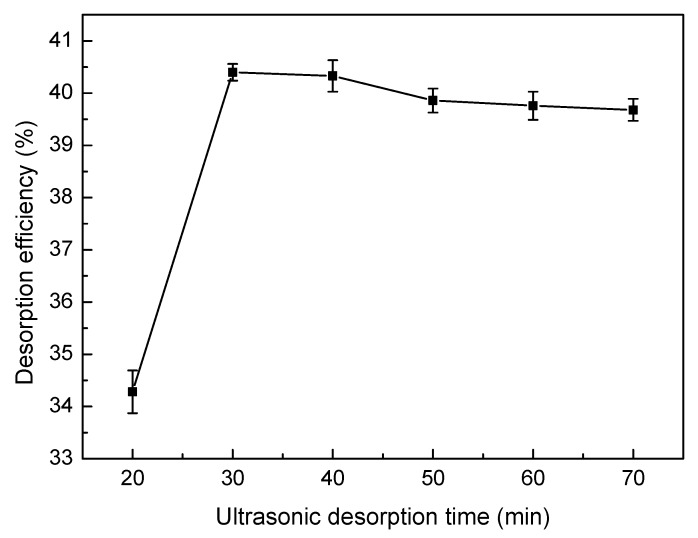
Effect of ultrasonic desorption time on desorption efficiency. (Desorption experiments—sample dosage: 0.05 g; desorption agent concentration: 0.4 mol/L; desorption temperature: 40 °C; desorption time: 20–70 min).

Fourth, Figure 16 illustrates the effects of ultrasonic desorption time on Ni(II) adsorption of the SLT nanocomposite. As seen, desorption efficiency increased during the first stage, with nearly no further increase occurring with an increase in sonication time. This phenomenon was responsible for the ultrasound rules of producing holes, that is to say, the reduction in the formation of hydroxyl radicals during the ultrasonic desorption process of aqueous by sonication cavitation. This process is mainly comprised of the formation, growth, and collapse by violent implosions to release pressures at local hot spots in aqueous solution [39]. In contrast, chemical adsorption such as electrostatic attraction, ion exchange, coordination and chelation between Ni(II) and the activated sites on the surface of the SLT nanocomposite restrained the desorption process. Comparatively, the high desorption equilibrium of Ni(II) on the adsorbent was reached at an ultrasound desorption time of 30 min.

Finally, to evaluate the reusability of the SLT nanocomposite, consecutive adsorption/desorption processes were performed five times. The effects of the regenerative cycles on the Ni(II) adsorption/desorption capacity and desorption efficiency are shown in Table 4. According to the results, it was clear that when retaining optimal adsorption/desorption conditions, the fading rate of the SLT nanocomposite of Ni(II) solution was almost unaffected even after the fourth run with the regenerated adsorbent, which was found to be 830.65 mg/g of the adsorption capacity, 187.91 mg/g of the desorption capacity, and 22.62% of the desorption efficiency, respectively. The experimental results indicated that the SLT nanocomposite exhibited a marked extent of regeneration ability. 

### 3.9. Adsorption Mechanism

**Figure 17:**
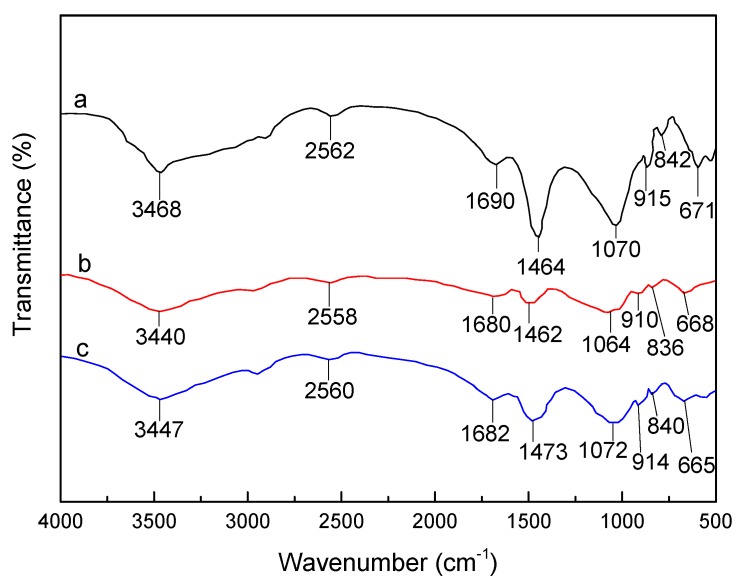
FTIR spectra of (**a**) SLT; (**b**) after adsorption Ni(II); and (**c**) after desorption Ni(II).

The FTIR spectra of (a) pure SLT, (b) Ni(II)-loaded SLT and (c) recovered SLT are shown in Figure 17. The bands at 3468 cm^−1^ of SLT were attributed to the intramolecular O–H stretching vibration absorption peak, as well as the characteristic absorption band of intermolecular hydrogen bonding between the phenol and alcohol molecules, weakened and shifted to a lower wavenumber at 3440 cm^−1^ after the adsorption of Ni(II), indicating that some of the O–H and corresponding hydrogen bonds interacted with Ni(II). Subsequently, this band slightly weakened after desorption. The characteristic adsorption band at 1690 cm^−1^ of the SLT corresponding to the asymmetric stretch vibration of the C=O bond even disappeared after adsorption of Ni(II), and rose again after desorption of Ni(II) at a lower wavenumber of 1682 cm^−1^. The vibration absorption peak of the carboxyl O–H bond located at 1464 cm^−1^ in the SLT, which apparently receded after Ni(II) adsorption, appeared at 1473 cm^−1^ after desorption of Ni(II). Moreover, the second –OH group in the carboxylic acid adsorption at 1070 cm^−1^ of the SLT faded at 1064 cm^−1^ after adsorption, then returned to 1072 cm^−1^ after desorption. The absorption bands at 915 cm^−1^ and 842 cm^−1^ in the SLT, which represented the stretching vibration absorption of the aromatic and phenol C–H stretching bond and Si–O bending vibration absorption, receded and moved to a lower wavenumber after Ni(II) absorption, then shifted back down to 914 cm^−1^ and 840 cm^−1^ after desorption. Furthermore, the characteristic S–H, C–S stretching vibration adsorption bands around 2562 cm^−1^ and 671 cm^−1^ were impaired after Ni(II) adsorption, which were then regained at 2560 cm^−1^ and 665 cm^−1^ after desorption. Based on the above-mentioned results, it was tentatively concluded that protons of the hydroxyl, carboxyl, and sulfhydryl activated groups of SLT were replaced by Ni(II) after adsorption. Overall, slight changes were observed in the FTIR spectra of the Ni(II)-loaded-SLT nanocomposite, and were basically restored to their original shape after desorption. Therefore, the basic structure and properties of the SLT remained relatively stable in the process of Ni(II) adsorption and desorption, signaling that it is a novel and highly-efficient renewable adsorbent.

**Figure 18:**
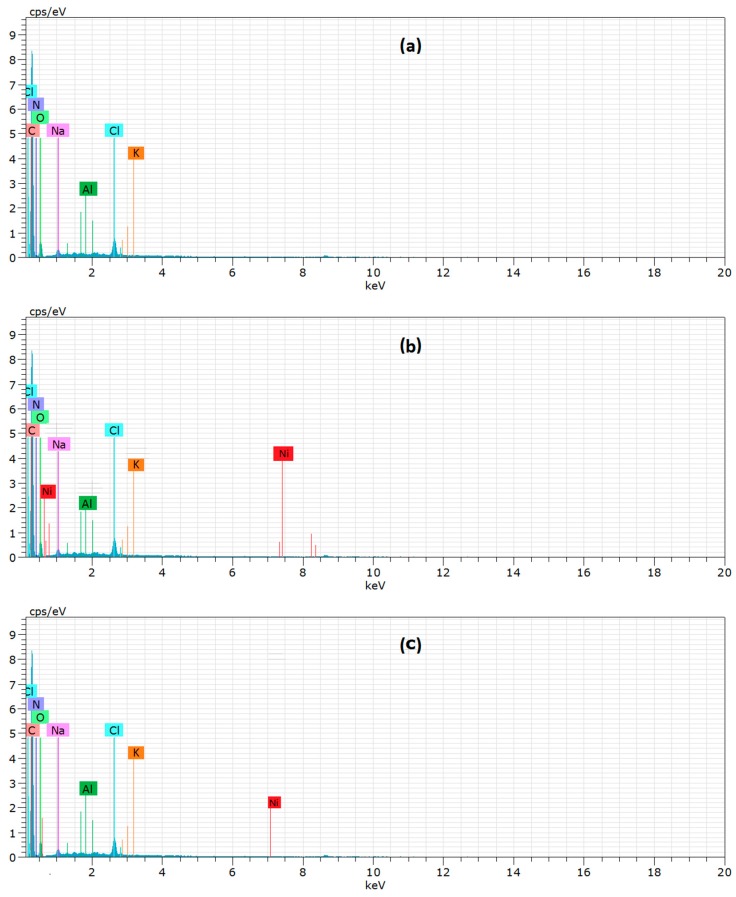
**Figure** **18.** Energy-Dispersive X-Ray (EDX) spectra for (**a**) SLT; (**b**) after adsorption Ni(II); and (**c**) after desorption Ni(II).

EDX is an analytical technique used for elemental analysis. EDX analysis of the SLT nanocomposite was recorded to confirm the existence of Ni(II) on the SLT after the adsorption experiments. Figure 18 presents the EDX spectra of the pure SLT (Figure 18a), the Ni(II)-loaded-SLT (Figure 18b), and the recovered SLT nanocomposite (Figure 18c). In the EDX spectrum of the SLT (Figure 18a), two new peaks of Ni(II) were found in the Ni(II)-loaded-SLT, verifying the presence of Ni(II) ions. After desorption, the content of Ni(II) was decreased (Figure 18c). The conclusion illustrated the interaction between the corresponding functional activated groups and Ni(II) ions, which was further confirmed by the following FTIR analysis of the adsorption mechanisms.

## 4. Conclusions

A novel SLT nanocomposite was prepared by intercalation. SLT can be effectively applied in the adsorption of Ni(II) ions from aqueous solutions. The maximum adsorption capacity with 0.05 g of SLT for Ni(II) reached 1134.08 mg/g under the optimal conditions corresponding to an initial Ni(II) concentration of 700 mg/L, pH of 5.5, adsorption temperature of 50 °C, and adsorption time of 40 min. The Ni(II) adsorption capacity significantly decreased with the increase in ionic strength. The adsorption kinetics and isotherms were well fitted to both the pseudo-second-order adsorption kinetics equation (*R*^2^ = 0.9991) and Langmuir isothermal adsorption models (*R*^2^ = 0.9976); these results indicated that the adsorption equilibrium was mainly dominated by monolayer chemical adsorption in the experimental range. 

The effects on the desorption capacity of the Ni(II)-loaded-SLT were observed by using HNO_3_ as a desorption agent in the ultrasonic oscillation treatment. The optimum conditions of desorption were as follows: the concentration of HNO_3_ was 0.4 moL/L, the temperature of desorption was 40 °C, and the time of ultrasonic desorption was 30 min. Under the optimum conditions, the maximum desorption capacity was determined as 458.21 mg/g and the optimal desorption efficiency was 40.40%.

The adsorption/desorption experiments demonstrated that the adsorption, desorption capacity, and desorption efficiency of SLT remained at a relatively high level after four rounds of adsorption/desorption recycling. The study showed that the SLT nanocomposite is an excellent potential adsorbent for the removal of Ni(II) from aqueous solutions, and can be regenerated and reused.

## Figures and Tables

**Table 1:** *R*^2^ and constant values for the different adsorption kinetics models of Ni(II). (Adsorption experiments—sample dosage: 0.05 g; initial Ni(II) concentration: 700 mg/L; pH value: 5.5; temperature: 50 °C; adsorption time: 40 min).

Metal	Parameters	Pseudo-First-Order		Pseudo-Second-Order		Intraparticle Diffusion		Elovich Model	
Ni(II)	*R*^2^	0.9883		0.9991		0.6781		0.7025	
	Constants	*k*_1_	0.0059 min^−1^	*k*_2_	0.0779 min^−1^	*k_i_*	9.274 mg/(g min^0.5^)	*α*	22.51 mg/(g min)
		*q_e_*	943.02 mg/g	*q_e_*	1109.84 mg/g			*β*	0.0097 g/mg

**Table 2:** *R*^2^ and constant values for the different adsorption isotherm models of Ni(II). (Adsorption experiments—sample dosage: 0.05 g; initial Ni(II) concentration: 700 mg/L; pH value: 5.5; temperature: 50 °C; adsorption time: 40 min).

Metal	Parameters	Langmuir		Freundlich		Temkin		Dubinin–Radushkevichl	
Ni(II)	*R*^2^	0.9976		0.9364		0.7522		0.6917	
	Constants	*K_L_*	0.0173 L/mg	*K_f_*	90.64 L/g	*b_t_*	40.87 J/mol	*B*	1.374 × 10^−8^ mol^2^·^2^
		*R_L_*	0.165						
		*q*_max_	1120.33 mg/g	1/*n*	0.139	*a_t_*	2.061 × 10^9^ L/g	*q*_max_	815.32 g/mg

**Table 3:** *q*_max_ value for the adsorption of Ni(II) on different adsorbents.

Adsorbents	*q*_max_ (mg/g)	Reference
SLT nanocomposite	1134.08	This paper
m-CTS/PVA/Ni(II)s composite	500	[31]
nano-hydroxyapatite/alginate composite	360	[32]
GC nanocomposite	228	[33]
Fe_3_O_4_-NH_2_ nanoparticle	232.51	[34]
GO-DPA composite	180.893	[35]
HCX	114.29	[36]
LNC/MMT	94.86	[17]
chitosan-MOF composite	60	[10]
Fe_3_O_4_-MnO_2_ nanoplates	55.63	[37]
Ni(II)-IIP polymer	12.96	[38]

**Table 4:** SLT adsorption/desorption capacities and desorption efficiency for Ni(II) after multiple cycles.

Recycle Times	First	Second	Third	Fourth	Fifth
**Adsorption *q_e_* (mg/g)**	1134.08	1104.79	1006.51	830.65	422.13
**Desorption *q_e_* (mg/g)**	458.21	441.06	297.23	187.91	76.04
**Desorption Efficiency (%)**	40.40	39.92	29.53	22.62	18.01
